# A Smartphone App and Personalized Text Messaging Framework (InDEx) to Monitor and Reduce Alcohol Use in Ex-Serving Personnel: Development and Feasibility Study

**DOI:** 10.2196/10074

**Published:** 2018-09-11

**Authors:** Daniel Leightley, Jo-Anne Puddephatt, Norman Jones, Toktam Mahmoodi, Zoe Chui, Matt Field, Colin Drummond, Roberto J Rona, Nicola T Fear, Laura Goodwin

**Affiliations:** 1 King's Centre for Military Health Research Institute of Psychiatry, Psychology & Neuroscience King's College London London United Kingdom; 2 Department of Psychological Sciences University of Liverpool Liverpool United Kingdom; 3 Academic Department of Military Mental Health Institute of Psychiatry, Psychology & Neuroscience King's College London London United Kingdom; 4 Department of Informatics King's College London London United Kingdom; 5 Department of Psychological Medicine Institute of Psychiatry, Psychology & Neuroscience King's College London London United Kingdom; 6 UK Centre for Tobacco and Alcohol Studies Department of Psychological Sciences University of Liverpool Liverpool United Kingdom; 7 Addictions Department Institute of Psychiatry, Psychology & Neuroscience King's College London London United Kingdom; 8 South London and Maudsley NHS Foundation Trust London United Kingdom

**Keywords:** behavior change techniques, smartphone, alcohol misuse, binge drinking, text messaging, ex-serving, armed forces, mobile phones

## Abstract

**Background:**

Self-reported alcohol misuse remains high in armed forces personnel even after they have left service. More than 50% of ex-serving personnel meet the criteria for hazardous alcohol use; however, many fail to acknowledge that they have a problem. Previous research indicates that interventions delivered via smartphone apps are suitable in promoting self-monitoring of alcohol use, have a broad reach, and may be more cost-effective than other types of brief interventions. There is currently no such intervention specifically designed for the armed forces.

**Objective:**

This study sought to describe the development of a tailored smartphone app and personalized text messaging (short message service, SMS) framework and to test the usability and feasibility (measured and reported as user engagement) of this app in a hard-to-engage ex-serving population.

**Methods:**

App development used Agile methodology (an incremental, iterative approach used in software development) and was informed by behavior change theory, participant feedback, and focus groups. Participants were recruited between May 2017 and June 2017 from an existing United Kingdom longitudinal military health and well-being cohort study, prescreened for eligibility, and directed to download either Android or iOS versions of the ”Information about Drinking for Ex-serving personnel” (InDEx) app. Through the app, participants were asked to record alcohol consumption, complete a range of self-report measures, and set goals using implementation intentions (if-then plans). Alongside the app, participants received daily automated personalized text messages (SMS) corresponding to specific behavior change techniques with content informed by the health action process approach with the intended purpose of promoting the use of the drinks diary, suggesting alternative behaviors, and providing feedback on goals setting.

**Results:**

Invitations to take part in the study were sent to ex-serving personnel, 22.6% (31/137) of whom accepted and downloaded the app. Participants opened the InDEx app a median of 15.0 (interquartile range [IQR] 8.5-19.0) times during the 4 week period (28 days), received an average of 36.1 (SD 3.2) text messages (SMS), consumed alcohol on a median of 13.0 (IQR 11.0-15.0) days, and consumed a median of 5.6 (IQR 3.3-11.8) units per drinking day in the first week, which decreased to 4.7 (IQR 2.0-6.9) units by the last week and remained active for 4.0 (IQR 3.0-4.0) weeks.

**Conclusions:**

Personnel engaged and used the app regularly as demonstrated by the number of initializations, interactions, and time spent using InDEx. Future research is needed to evaluate the engagement with and efficacy of InDEx for the reduction of alcohol consumption and binge drinking in an armed forces population.

## Introduction

Alcohol misuse is common in the United Kingdom (UK) armed forces and the prevalence is higher in the military than in the general population [1-3], with the trend continuing after they leave service [1]. More than 50% of those who have left military service meet the criteria for hazardous alcohol use, defined as scoring 8 or more on the alcohol use disorders identification test (AUDIT; [4]). This prevalence rate is almost double of that found in the general population [5]. Additionally, 47% of ex-serving personnel report binge drinking, defined as 6 or more units for females and 8 or more units (1 UK unit=8g ethanol) for males, per session at least once per week [3].

Most people in the general population underestimate their drinking and do not perceive it as problematic, even when the level of consumption is potentially harmful to health [6]; young men are at particular risk of underestimating their drinking [6]. This pattern is similar among armed forces personnel with less than half of hazardous drinkers recognizing that they have an alcohol problem and seeking medical help [7]. There is a culture of heavy alcohol use in the armed forces, which may be encouraged or maintained by social determinants [8]; therefore, leaving service could provide an opportunity to initiate behavioral change in settings with less peer pressure to conform to social norms.

In the last decade, computer and Web-based interventions (eg*,* Down Your Drink [9]) have been harnessed to increase reach, provide real-time monitoring, and offer personalized delivery [9-11]. More recently, the mode of intervention delivery has shifted from Web-based to mobile-based [12]. Mobile apps for use in health have proven to be an effective and successful method of providing patient-centric interventions that are based on real-time data and needs [13].

There are a large number of alcohol-related apps available to the general population with a recent content analysis identifying more than 600 apps, of which 91 were identified as focusing on alcohol reduction [14]. It has been reported that many apps lack an evidence base and make no reference to the scientific literature [14,15]. Recent research has found the use of mobile apps as brief alcohol interventions to be effective compared with traditional delivery methods (eg*,* face-to-face) [16,17]; however, the content of most existing alcohol smartphone interventions is based on public health guidelines regarding safe alcohol limits [14,18]. These alcohol limits may not be *perceived* as credible because they are viewed as state sponsored and are often at odds with individual beliefs, prevailing social context, and perceptions of consumption [18-21]. Many users do not maintain engagement with mobile health interventions [22]. Further, the majority of existing alcohol mobile apps emphasize long-term health consequences which are seen as remote risks, especially by young drinkers [15,17,23]. A recent meta-analysis suggests that it may be more effective to focus on short-term detrimental consequences to encourage individuals to reduce their alcohol consumption [24].

Most existing alcohol apps include self-monitoring (eg*,* Drink Less [23], Drink Aware [25], One You Drinks Tracker [26]), wherein users are encouraged to regularly record and monitor (via visual graphics) their alcohol consumption within an app [23,27]. Self-monitoring was found to be the most effective behavior change technique (BCT) for reducing alcohol use; a BCT is defined as a specific, irreducible component of an intervention designed to change behavior and a putative active ingredient in an intervention [28]. A recent review of computer and Web-based delivered alcohol interventions suggested that provision of normative feedback, goal review, and inclusion of the social norms approach in combination were associated with better outcomes [24]. There is also evidence that short message service (SMS) text message interventions can be successful in encouraging people to change their behavior [29,30], and further benefits may be gained by incorporating user input to tailor the SMS text messages. However, to the authors’ knowledge, there is no published work that seeks to develop an alcohol reduction app for ex-serving personnel.

We are not aware of any mobile health app that seeks to customize a brief alcohol intervention using personalized SMS text messages. In this study, we describe the development of the “Information about Drinking for Ex-serving personnel” (InDEx) mobile phone app, a tailored 4 week (28 day) intervention specifically designed to target ex-serving personnel who meet the criteria for hazardous alcohol use, which is likely to impact on their functioning. The purpose of this study was to design an engaging, responsive, and usable smartphone app that delivers personalized SMS text messages and gathers alcohol usage data and to test the usability and feasibility, measured and reported as user engagement, of this app in a hard-to-engage ex-serving population. Our primary outcome measure was adherence with InDEx, which was measured by the number of weeks participants engaged with the app. Our secondary outcome measures were how many times participants used the app (eg*,* utilization of the drinks diary) and the proportion of participants using InDEx at the end of the study period.

## Methods

### Participants

Potential participants were eligible for inclusion if they had served in the UK military, were aged 18-65 years, owned an iPhone or Android device released after 2012, were willing to receive daily SMS text messages, currently resided in UK, and were capable of providing informed consent. Those who had an AUDIT score lower than 8 or greater than 19 were excluded because InDEx is focused on intervening among those drinking hazardously or harmfully, who are likely to be experiencing short-term consequences of their drinking, yet unlikely to be seeking any treatment for this misuse. Those scoring above 20 on the AUDIT meet criteria for probable alcohol dependency and we felt that they may require more intensive treatment. Potential participants took part in the King’s Centre for Military Health Research cohort study [2,31] and consented to receive further contact. Participants were asked to use the InDEx app for a period of 4 weeks (28 days) between May 2017 and June 2017. Providing informed consent, downloading the app, and registering an account constituted enrollment in the study. Participants were compensated £40 for their time.

### App Design and Development

Design and development of the InDEx app was undertaken on an Apple MacBook Pro, 2.5 GHz i5 Intel processor and 8GB RAM. Drifty Co IONIC Framework version 1 [32] was used as the cross-platform framework to enable iOS and Android deployments using Atom [33] as the development environment (see Multimedia Appendix 1 for an infographic of the InDEx ecosystem).

A full description of the development process, including the InDEx app source code, is available in [34]. A summary is provided hereafter.

#### Specification and Development

The development of the InDEx app was academic-led and supported by experts in smartphone app development, epidemiology, addiction psychiatry, and military mental health. The content of the intervention incorporated effective components of previous electronic alcohol interventions (eg, [24]) with SMS text messages informed by the health action process approach (HAPA). HAPA theorizes that individuals work through a number of stages to change their behavior, emphasizing the motivational processes underpinning behavioral intentions and the various processes that bring about behavior change [35,36]. The delivery was split into 3 stages, based upon the HAPA model, with the content of the app and SMS text messages corresponding to each stage, for example, goal setting was only introduced at stage 2 (and available for use in stage 3). The stages were:

- Stage 1: Normative feedback (defined below), action self-efficacy, and self-monitoring
- Stage 2: Maintenance of self-efficacy and action planning
- Stage 3: Recovery of self-efficacy and coping planning

The features were grouped into the following modules:

1. Account Management: Participants can modify personal information (eg, first name, last name, and mobile number), password, and app parameters (eg, automatic log-out and clear local storage).
2. Assessment and Normative Feedback: Captures the participant’s response to a set of questions (defined by the research team) and aggregates responses to produce an infographic representing the participant’s alcohol consumption in comparison to the general population.
3. Self-monitoring and Feedback: Records alcohol consumption by participants and provides a range of visual (eg, charts, figures, and text) metrics to allow for monitoring of consumption.
4. Goal (setting and review): Participants can set goal(s) based on the implementation intentions [37] methodology; visual feedback provides feedback on progress toward achieving goal(s) set.
5. SMS Text Messaging (review): Provides a facility to review SMS text messages sent to and from the InDEx central server system. Further, participants can rate automated SMS text messages (5 star Likert rating).

The app was developed using Agile development methodologies [38] in which an incremental design approach is employed and each increment builds upon the functionality of the previous. Each increment underwent rigorous testing by stakeholder and expert participants sourced from King’s Centre for Military Health Research and University of Liverpool (n=17) to ensure software quality and usability. Stakeholders and expert participants were requested to provide feedback on usability, language, functionality, and errors at each increment point. The development cycle did not progress until functionality and source errors were addressed.

To create an account, a participant was required to provide their first name, last name, email address, mobile telephone number, username, password, and in-app informed consent. All sensitive information such as password was encrypted using Bcrypt hashing algorithms (salt factor 10).

**Figure 1:**
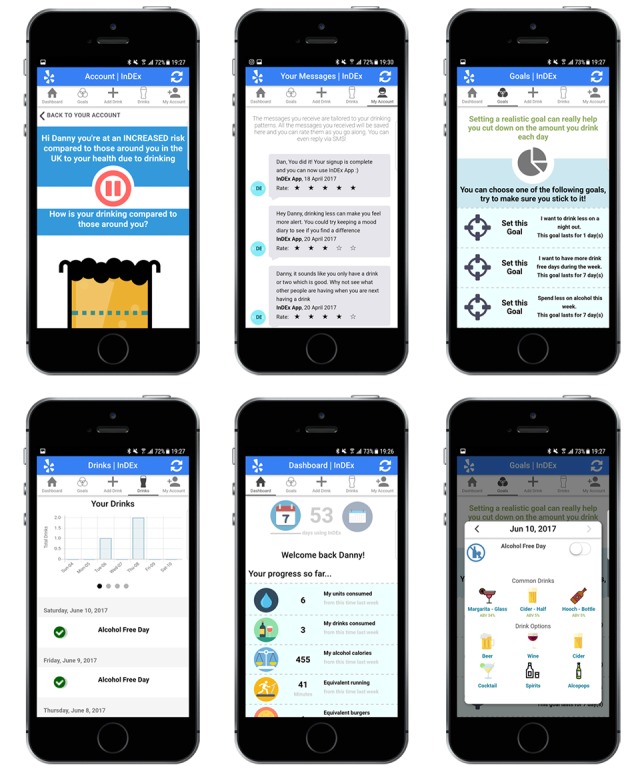
Example screenshots of interactions with the InDEx app (left to right, beginning at top): normative feedback, personalized text message history, set a goal, drink diary, dashboard and add a drink. Source: King’s Centre for Military Health Research, King’s College London.

InDEx app is presented in Figure 1. The app was designed with limited storage capabilities to avoid concerns regarding confidentiality and privacy of data. Only the username and a secure JSON Web Token denoting the user’s time restricted session were stored on the local device with all other data being stored in temporary memory and accessible via application programming interface calls. The app was also available for limited offline use.

#### Operating System Selection

In UK, 4 out of 5 adults own a smartphone; among 18-44-year-olds, adoption is higher at 91% [39] with the majority (over 90%) of smartphones operating either on Google Android or Apple iOS. Based on this information, InDEx was developed for use with both Google Android and Apple iOS enabled devices ensuring that a wide spread of participants could be included.

#### Personalized Text Messaging

The InDEx app was complemented by tailored SMS text messaging that provides prompts to use the drinks diary, suggests alternative behaviors, and provides feedback on goals. A bank of 180 tailored SMS text messages was developed in line with delivery stages (defined earlier), which were informed by the HAPA framework and from discussion groups with ex-serving personnel to further refine the messages (Table 1). Each message had the following characteristics: what day it would be sent, message content, and a decision tree defining when it should be triggered. A participant would receive at least one SMS text message each day, up to a maximum of 2. The ultimate design and objective of each message was to prompt diary completion and to suggest alternative behavior related to their individual alcohol consumption.

InDEx uses baseline and contiguous measurements to inform the type of SMS text messages a participant receives to provide a participant-centric approach. Baseline measurements are used to identify suitable messages and as a participant engages with InDEx, continuous measurements are used to reflect current behavior and attitude; for example, if a participant reports feeling depressed or anxious (measured by the patient health questionnaire [40]), a message with suggestions for alternative behaviors to cope with these symptoms (eg, going for a walk) is sent. The messages covered a wide range of topics to target beliefs and motivations with the primary aim of increasing the participant’s awareness of their drinking habits and behaviors. The messages were divided into 3 categories: (1) tailored: personalized to drinking habits, baseline, and weekly measurements; (2) tailored and triggered: tailored to baseline and contiguous measurements and a specific event occurring; and (3) targeted (generic): sent on specific days to highlight inactivity, a new feature, or to remind users about an issue. See Table 1 for examples of SMS text messages. The message bank and decision tree for sending SMS text messages are available upon request from the corresponding author.

SMS text messages and two-factor authentication codes (used to verify the participant’s mobile phone number) were sent automatically using Twilio’s Application Programming Interface via InDEx central command servers. No human involvement was required. All SMS text messages sent to participants were visible in the app (“My Messages” page). Participants could rate any message (rating scale 1-“poor” to 5-“excellent”) and provide SMS text message responses, which were stored and displayed to the user but not monitored by the study team.

#### Submission and Testing

InDEx was submitted to the Google Play and Apple iTunes App stores via Google Play Developer Console and Apple iTunes Connect, respectively. For testing of InDEx, a private testing group was created; only those who had been given permission were able to access and download InDEx.

### Measurement Reporting

**Figure 2:**
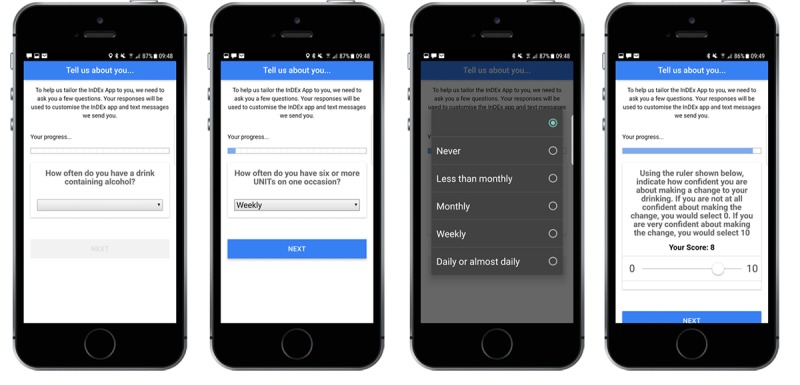
Example screenshots of the InDEx app measurement (questionnaire) module. Source: King’s Centre for Military Health Research, King’s College London.

All measurements were collected via the modules, as seen in Figure 2. The study team had no ability to modify or influence any measurement response.

Upon successful registration (referred to as “day 0”), participants completed several baseline questionnaires that collected the following information: (1) Age and sex; (2) Alcohol consumption and alcohol use disorders via alcohol use disorders identification test (AUDIT; [41]); (3) Symptoms of anxiety using the two item Generalized Anxiety Disorder Scale (GAD-2; [42]); (4) Symptoms of depression using the two item patient health questionnaire (PHQ-2; [40]); (5) Symptoms of Post-Traumatic Stress Disorder were assessed using the five item Diagnostic and Statistical Manual of Mental Disorders Post-Traumatic Stress Disorder Scale [43]; and (6) Readiness to Change and Self-efficacy Scales (score range: 0-10) [44].

Baseline measurement responses informed the type of SMS text message a participant would receive. Although this was optional, the baseline measures were asked again upon completion of the study (day 28).

#### Weekly Measurements

Participants were asked on days 8, 15, and 22 to complete GAD-2, PHQ-2, and Readiness to Change and Self-Efficacy Scales. Any response provided by the participant further informed the tailoring of the SMS text messages, for example, a participant who scores low on the Readiness to Change Scale is sent supportive messages to encourage a willingness to change.

#### Reporting Alcohol Consumption

Participants could “*record”* alcohol beverage(s) or an “*alcohol free day* ” via the *“Add Drinks”* tab; Multimedia Appendix 2 illustrates the types of alcoholic drink a participant could record. Self-reported alcohol consumption is a standard method for assessing the efficacy of low-intensity interventions [14,17,23].

**Table 1:** An example of the type of *personalized* SMS text messages sent to an individual throughout their use of the app.

Day to be sent	Type	Related BCT^a^	Message
3	Tailored	Mental rehearsal of successful performance (BCT 15.2)^b^	Hi {name}, try thinking that if I am at the pub this week and feel like drinking then imagine how fresh I will feel the next day if I do not drink a lot.
8, 14, 21, 28	Tailored and triggered	Self-monitoring of behavior (BCT 2.3)^b^	Hi {name}, have you logged your drinks from last week? It’s quick and easy to do, just go onto the “drinks” tab in the app.
8	Generic	Action planning (BCT 1.4)^b^	Hi {name}, why not set a goal to reduce the amount you drink? It has been found to really help reduce your drinking, you can start now by clicking on the “goals” tab in the app.

^a^BCT: behavior change technique.

^b^*Personalized* SMS text fields with reference to relevant *behavior change technique taxonomy.*

Participants could optionally provide volume, strength, price, and calories; however, if no information was provided, UK standard data were used [45]. Further, participants could record who they were drinking with, where they were drinking, and, if consent was provided, their geographical position was recorded.

#### Engagement and Usability

We measured usability by frequency of engagement using a published procedure [46] that included the number of times the app was initialized (ie, started when not running in the background), the average session duration (ie, time spent using the app and overall and for each page), the number of times a participant performed an interaction (ie, synchronized data, added a drink, and added a SMS text message rating), and the number of weeks in which participants remained engaged with the app. User engagement was defined as having at least 3 client-server interactions in a 7 day period, other than receiving a SMS text message, and was used as a proxy for usability.

Participant engagement was tracked using Google Analytics for Mobile which recorded data when the participant was online or offline. It was not possible to confirm and track if a participant read the SMS text messages, except in cases where the participant provided a rating from within the app.

#### Clinical Monitoring and Risk Management

Prior to the study commencing, a risk protocol was developed and approved by the University of Liverpool Ethics Committee. Adverse health events were ascertained via automatic monitoring and reporting based on measurement responses and alcohol consumption. A clinician received all warning notifications, which were predefined by the research team for review. If the clinician felt that the event was clinically significant, participants were offered a call by a clinician (for those who declined, a reason was recorded) to discuss the adverse health event. All participants, irrespective of an adverse health event, were provided with a signposting and pathways to local support and assistance via a “Support” page within the app.

### Data Analysis

We calculated descriptive statistics to estimate engagement and usability with the app, which were used as a proxy for the feasibility of the InDEx app (to address the primary outcome). Engagement statistics were reported as median and interquartile range (IQR) because the data were not normally distributed (evaluated using skewness and kurtosis values and visualizing the data). Popularity of pages was inferred from the summation of the total number of times each page was viewed by users, and pages were then ranked from highest to lowest number of views.

The average number of drinking days, drink free days, units consumed, units consumed per drinking day, and alcoholic drinks per drinking day were computed across participants and reported as median and IQR. In this study, the number of binge drinking days was computed per week based on the number of days participants reported consuming 6 or more alcoholic drinks (to address the secondary outcome). Self-reported baseline and weekly measurements were presented as median and IQR, except for Readiness to Change and Self-Efficacy Scales, which were presented as mean and SD. Analyses were undertaken using STATA SE 14.2.

### Ethical Approval

Ethical approval was obtained from the local Research Ethics Committee at the University of Liverpool (reference: #0625).

## Results

### Recruitment, Study Enrollment, and Participant Demographics

**Figure 3:**
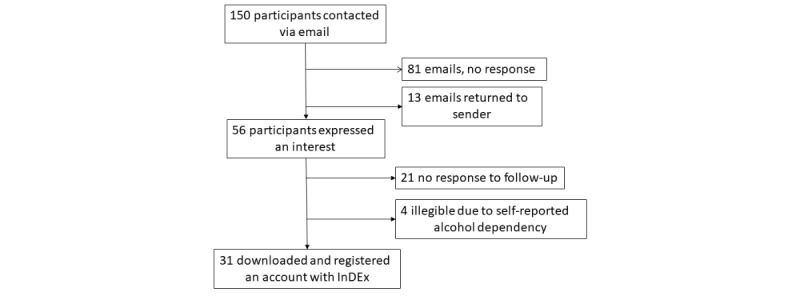
Participant flow through the study.

As shown in Figure 3, 150 individuals were contacted via email to participate in this study; 13 emails bounced back as the email addresses were not valid. Overall, 22.6% (31/137) downloaded and registered an account with InDEx, 87% (27/31) male and 13% (4/31) female. Of those who joined, 16% (5/31) were aged 25-39 years, 19% (6/31) were aged 40-44, 19% (6/31) were aged 45-49, 19% were aged 50-54 (6/31), and 26% (8/31) were aged 55-64. Finally, 84% (26/31) reported serving in the military for 12 years or more.

### Engagement

Participants used the InDEx app for a median of 4.0 (IQR 3.0-4.0) weeks (primary outcome), initializing 15.0 (IQR 8.5-19.0) times over 4 weeks and engaging in 29.0 (IQR 20.0-40.5) sessions for a median of 48.8 seconds (IQR 35.1-73.1). Table 2 provides the engagement measures relating to the level of engagement and adherence; 74% (23/31) of participants used the app every week (maximum 4 weeks) with 87% (27/31) using the app in the final week. Table 3 describes the top 10 pages viewed by participants with the “Dashboard” (38.41%) page being the most popular.

### Drinking Behaviors

Table 4 describes the frequency with which participants made a diary entry. Participants consumed alcohol a median of 13.0 (IQR 11.0-15.0) days, had 15.0 (IQR 13.0-17.0) drink free days, and recorded 2.0 (IQR 1.0-4.0) alcoholic drinks per drinking day with a median of 4.7 (IQR 2.3-9.1) units per day.

Table 5 illustrates the drinking behavior of participants over the study period. During week 1, participants reported a median of 2.0 (IQR 1.0-3.0) binge drinking days per week with a similar result in week 4.0 (2.0; IQR 1.0-2.5). However, reductions in units per drinking day from week 1 (5.6; IQR 3.3-11.8) to week 4 (4.7; IQR 2.0-6.9) and units consumed (week 1: 22.9; IQR 14.3-32.4 and week 4: 15.9; 11.6-26.9) was observed.

### Measurement Responses

Table 6 summarizes participants’ baseline and weekly self-reported measurement responses. Participants had a baseline median AUDIT score of 11 (IQR 10-12), indicating hazardous alcohol use, with an average Readiness to Change Scale score of 4.4 (SD 3.2), indicating some willingness to change. A small change in AUDIT score was observed for participants who self-reported for Day 0 (registration) and Day 28 (final day) based on median score; however, they would still be classified as hazardous drinkers. Most participants did not report anxiety or depression symptoms (measured via GAD-2 or PHQ-2) throughout the study.

### Text Messaging

In total, 1083 (mean 36.1, SD 3.2) SMS text messages were sent. Participants were able to reply to messages but were informed that responses would not be monitored. There were 18 replies and 42 SMS text message ratings. The mean rating of content suitability was 2.5 (SD 1.3), indicating a neutral rating for the content of those messages. One participant withdrew consent for receiving SMS text messages on day 16 of the study.

**Table 2:** Engagement measures over the study period per participant.

Engagement Measure	Median (IQR^a^)
Initializations^b^	15.0 (8.5-19.0)
Session count	29.0 (20.0-40.5)
Session duration (s)	48.8 (35.1-73.1)
Interactions^c^	223.0 (182.3-303.5)
Weeks active	4.0 (3.0-4.0)

^a^IQR: interquartile range.

^b^App initialization reflects the app being opened without a background session existing.

^c^Defined as a participant performing a click event (eg*,* add drink, log-out, change page, change drinks diary chart).

**Table 3:** Top 10 viewed pages within the InDEx app visited by participants within the study period.

Page	n (%)
Dashboard	4045 (38.41)
Drinks diary	3031 (28.78)
Add drink	1160 (11.01)
Account	390 (3.70)
Goals	379 (3.59)
Normative feedback	244 (2.31)
Weekly assessment	166 (1.57)
Login	148 (1.40)
Support	102 (0.96)
Your messages	98 (0.93)
Other pages	766 (7.27)

**Table 4:** Number of drinking days, drink free days, units consumed, and alcoholic drinks per drinking day across the study period (4 weeks, n=31).

Reported alcohol consumption	Median (IQR^a^)
Drinking days	13.0 (11.0-15.0)
Drink free days	15.0 (13.0-17.0)
Units per drinking day	4.7 (2.3-9.1)
Units consumed	79.4 (58.4-117.3)
Alcoholic drinks per drinking day	2.0 (1.0-4.0)

^a^IQR: interquartile range.

**Table 5:** Drinking behavior of participants over the study period; n denotes number of participants who recorded an alcohol event during the period.

Reported alcohol consumption	Week 1 (n=31), median (IQR^a^)	Week 2 (n=30), median (IQR)	Week 3 (n=29), median (IQR)	Week 4 (n=31), median (IQR)
Drinking days	4.0 (3.0-5.0)	3.0 (3.0-4.0)	3.0 (3.0-4.0)	3.0 (2.0-3.0)
Drink free days	3.0 (2.0-4.0)	4.0 (3.0-4.0)	4.0 (3.0-4.0)	4.0 (4.0-5.0)
Units per drinking day	5.6 (3.3-11.8)	6.5 (2.3-9.1)	4.54 (2.3-8.9)	4.7 (2.0-6.9)
Units consumed	22.9 (14.3-32.4)	20.4 (14.6-25.0)	18.1 (12.7-26.3)	15.9 (11.6-26.9)
Alcoholic drinks per drinking day	2.0 (2.0-4.0)	3.0 (1.0-4.0)	2.0 (1.0-4.0)	2.0 (1.0-4.0)
Binge drinking days per week^b^	2.0 (1.0-3.0)	2.0 (1.0-2.0)	1.0 (0.0-2.0)	2.0 (1.0-2.5)

^a^IQR: interquartile range.

^b^Defined as having 6 or more alcoholic drinks in a session.

**Table 6:** Self-reported baseline and weekly measurement responses.

Variable	Day 0 (n=31)	Day 8 (n=25)	Day 15 (n=25)	Day 22 (n=21)	Day 28 (n=22)
Two item Generalized Anxiety Disorder Scale, median (IQR^a^)	0 (0-1)	0 (0-0)	0 (0-1)	0 (0-0)	0 (0-0)
Two item patient health questionnaire, median (IQR)	0 (0-2)	0 (0-0)	0 (0-1)	0 (0-0)	0 (0-0)
Alcohol use disorders identification test, median (IQR)	11 (10-12)	N/A^b^	N/A	N/A	10 (8-12)
Self-efficacy, mean (SD)	6.7 (2.7)	5.9 (3)	4.9 (3.2)	6.3 (2.5)	4.5 (3.1)
Readiness to change, mean (SD)	4.4 (3.2)	4 (3.3)	3.4 (2.8)	4.9 (3.2)	3.7 (2.7)

^a^IQR: interquartile range.

^b^N/A: not applicable.

## Discussion

### Principal Findings

The aim of this paper was to design an engaging, responsive, and usable smartphone app that delivered personalized SMS text messages and gathered alcohol usage data. We tested the usability and feasibility, measured and reported as user engagement, of this app in a hard-to-engage ex-serving population. The InDEx app was codesigned by stakeholders and ex-serving personnel, with the results indicating successful user engagement and adherence. Based on the primary and secondary outcome measures, the participants used the app for the length of the study period, with two-thirds of participants using the app every week and the majority still using it in the final week (27/31, 87%). These engagement measures suggest that participants were highly active in using InDEx during the study period and that it is feasible to collect alcohol consumption data from this population. On average, most participants reported drinking on just under half of the days in the study period with participants reporting binge drinking on average 2 times a week. Reductions in units per drinking day and units consumed per week were observed across this 4 week study (yet the average number of drinks remained consistent); however, it is not possible to determine whether this may be due to participants changing the size and alcohol content of their drinks in this small feasibility study.

In this study, the most frequently opened page was the “Dashboard,” the “Drinks Diary” page was the second most frequently accessed, and the “Add Drinks” page was third. The top 3 most viewed pages accounted for 78.20% (8236/10529) of all app views, indicating that most participants used the InDEx app primarily for monitoring drinks and the other features were not used as frequently. InDEx offered the ability to set a goal using an if-then format; however, participants used this feature rarely even after encouragement to set a goal via SMS text message and in-app prompts. This may be due to the sample not believing that they have a problem or being unable to navigate to and set a goal, which will be explored further in future work.

We applied behavior change theory [28] to create a smartphone app that incorporated a tailored SMS text messaging framework in an attempt to engage with users who are usually hard to reach [47-49]. It is difficult to ascertain if, and to what extent, SMS text messages encouraged alcohol reduction or app engagement. Future work is needed to assess the relationship between receiving a SMS text message and engagement with the app. The InDEx app takes advantage of a delivery method that circumvents practical and psychological barriers by utilizing digital technology. Participants were compensated for registering but had no financial incentive to use the app for the study period; nevertheless, they spent a median of 4 weeks engaging with the app.

InDEx has features not offered in other currently available alcohol apps [17,18,23]. First, it offers a user-centered and personalized design; features (ie, normative feedback) of the app were generated through codesign discussions with stakeholders and ex-serving personnel and developed using an iterative development framework to ensure that they were properly focused. The second major facet of the app was the use of BCTs in conjunction with data collected via the app to personalize the SMS text messages sent to participants. These features exploit contemporary technology which, as our feasibility study suggests, has the potential to promote the acceptability of InDEx and encourages users to engage with the app to record and thereby self-monitor their alcohol consumption. Third, InDEx is focused on reducing alcohol use among those meeting criteria for hazardous to harmful alcohol use (who may not recognize that they have a problem with alcohol), unlike other studies which have sought to support recovery for alcohol dependency (alcoholism) [21].

To the authors’ knowledge, this was the first study to use SMS text messages embedded in an app to specifically focus on improving engagement and alternative behavior related to the individual alcohol consumption of ex-serving personnel. Although several studies have sought to investigate the impact that SMS text messages and tailoring can have on adherence, the combined use of the 2 strategies within the framework of a mobile app has never been attempted before.

### Limitations

Notwithstanding the study strengths, our findings have some limitations. First, the baseline weekly alcohol consumption data were self-reported, albeit using reliable, consistent, and “gold standard” measurements. As with all self-report measures, recall and social desirability biases may have impacted responses to be more favorable than if collected using objective methods, such as transdermal alcohol monitoring [50,51]. Second, participants were asked to use the InDEx app for 4 weeks. Although the app appears feasible and acceptable to users based on engagement measurements during the study period, this study was not designed to ascertain the long-term benefits. Third, the sample size and design were appropriate for feasibility testing but not for assessing the efficacy of the app. Fourth, participants were recruited via the King’s Centre for Military Health Research and offered an incentive to take part, resulting in a possible selection bias because participants had consented to participate in a research study previously. Finally, we studied InDEx in isolation and did not directly compare it with other app-based interventions.

### Conclusions

In summary, the results of this study suggest that the InDEx app was feasible to implement and acceptable to participants, who typically engaged with the app for most of the study duration. It was feasible that participants reduced alcohol consumption during the study period, but this needs to be specifically addressed in a randomized controlled trial. Future research is needed to evaluate the engagement with and efficacy of InDEx for the reduction of alcohol consumption and binge drinking in an armed forces population.

## Appendix

Multimedia Appendix 1 Online supplement: Infographic representing the InDEx ecosystem.

Multimedia Appendix 2 List of alcohol types and categories included in the InDEx App.

## Abbreviations

AUDIT: alcohol use disorders identification test
BCT: behavior change technique
GAD: generalized anxiety disorder
HAPA: Health Action Process Approach
InDEx: Information about Drinking for Ex-serving
IQR: interquartile range
PHQ: patient health questionnaire
SMS: short message service
