# Supplementary figures and images for: A Smartphone App and Personalized Text Messaging Framework (InDEx) to Monitor and Reduce Alcohol Use in Ex-Serving Personnel: Development and Feasibility Study
Source: JMIR Mhealth Uhealth. 2018 Sep 11;6(9):e10074. doi: 10.2196/10074 (PMC6231744; doi:10.2196/10074)

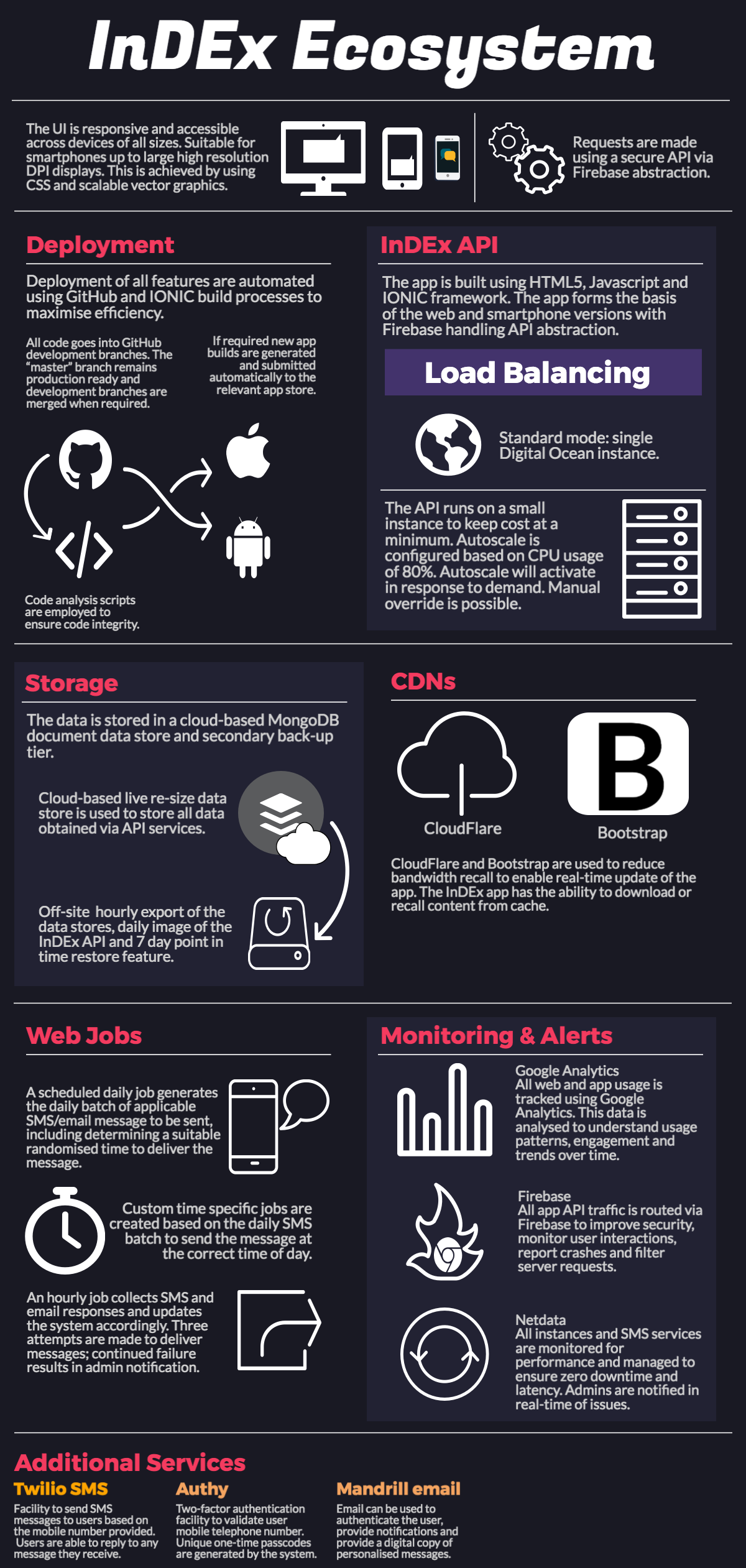

Supplement: Multimedia Appendix 1 [file mhealth_v6i9e10074_app1.png]
