# Supplementary material for: A Smartphone App and Personalized Text Messaging Framework (InDEx) to Monitor and Reduce Alcohol Use in Ex-Serving Personnel: Development and Feasibility Study
Source: JMIR Mhealth Uhealth. 2018 Sep 11;6(9):e10074. doi: 10.2196/10074 (PMC6231744; doi:10.2196/10074)
Supplement: Multimedia Appendix 2 [file mhealth_v6i9e10074_app2.pdf]

Multimedia Appendix 2

Alcohol Category	Alcohol Type (measure)
Beer	Beer (Pint/Half), Shandy (Pint/Half), Ale (Pint/Half), Bitter (Pint/Half)
Wine	Red (Large, Medium, Small), White (Large, Medium, Small), Rose (Large, Medium, Small), Prosecco (Glass)
Cider	Cider (Pint/Half)
Cocktail	Bloody Mary (Glass), Screwdriver (Glass), Martini (Glass), Margarita (Glass), Cosmopolitan (Glass), Mojito (Glass)
Spirits	Cognac (Single, Double), Gin (Single, Double), Rum (Single, Double), Vodka (Single, Double), Tequila (Single, Double), Liqueurs (Single, Double), Sambuca (Single, Double), Sours (Single, Double), Others (Single, Double)
Alcopops	WKD (Bottle), Hooch (Bottle), Smirnoff Ice (Bottle)
